# The Psychosocial Impact of COVID-19 on health care workers

**DOI:** 10.1590/S1677-5538.IBJU.2020.S124

**Published:** 2020-07-27

**Authors:** Bárbara Otonín Rodríguez, Tania Lorca Sánchez

**Affiliations:** 1 Clinical Psychologist Madrid Spain Clinical Psychologist, Madrid, Spain; 2 Private Consulting Madrid Spain Private Consulting, Madrid, Spain

**Keywords:** Occupational Health, COVID-19 diagnostic testing [Supplementary Concept], Stress Disorders, Post-Traumatic, Burnout, Psychological

## Abstract

At the end of 2019, a disease was identified, COVID-19, caused by a new type of easy and fast spreading virus, which led to the beginning of a worldwide pandemic. One of the most exposed groups to the virus and its psychosocial consequences is the healthcare workers, due to their implication in caring for affected people. Health workers are exposed to a fast and unpredictable situation that requires more human resources and materials than usual, however, the lack of means on account to this situation entails an increased probability of suffering different consequences, including the burnout syndrome, to which, generally, this professionals are already vulnerable. In addition, quarantine is added as a measure to prevent the spread of the pandemic, which is another handicap for healthcare workers. Quarantine means these professionals are more likely to suffer the foreseeable psychological consequences in general population, specifically, it has been observed that Post-Traumatic Stress Disorder (PTSD) is more prevalent, because of the stress load of the situation experienced.

## INTRODUCTION

A pandemic generates in human beings one of the most primitive answers at psychological level: fear. Fear is an emotion that allows us to react to a real or imagined event which we consider a threat, at the physical level as well as psychological or socioeconomic levels. Thus, fear guarantees our survival. As other emotions, fear activates the three levels of response in our body: cognitive, physiological and motor.

Fear is an emotion we experience as unpleasant, even though it is in and on itself functional, it becomes dysfunctional when it dominates our life, either our response exceeds the event we need to manage -our system generates a state of alert over something that could happen, anticipating and suffering the negative effects without actually happening-, or the event which we are exposed to is highly traumatic and exceeds our available resources, generating very high stress levels. This is when a disorder derived from anxiety occurs - generalized anxiety disorder, panic disorder, agoraphobia- or stress -post-traumatic stress disorder (PTSD), complex PTSD, prolonged grief disorder. When a situation of fear and distress lasts, high levels take time to disappear. If we add other factors, such as loss of health, a loved one, job position or quarantine, post traumatic effects may last.

Although anxiety, depressive and distress symptoms can be found in high levels in general population, some groups can be more vulnerable than others to the psychosocial effects of pandemics. These would be people who contract the disease, those with high infection risk; people with preexisting medical, psychiatric, or substance use problems are at increased risk for adverse psychosocial outcomes; and, especially health care providers. This last group, considered essential workers, becomes both directly exposed to virus and to the psychosocial consequences derived from its propagation. They are particularly vulnerable, given their risk of exposure to the virus, concern about infecting and caring for their loved ones, shortages of personal protective equipment (PPE), longer work hours, and involvement in emotionally and ethically fraught resource-allocation decisions (1).

In fact, early research conducted on Chinese population shows that a significant proportion of health workers have depression symptoms (50.4%), anxiety (44.6%), insomnia (34%), discomfort (71.5%) (2). This evidence makes us consider highly relevant to focus on this population.

In this chapter we will describe the possible consequences at the psychological level in sanitary groups considering the different factors that affect the way they are facing this pandemic crisis.

### Psychological consequences in healthcare workers

#### Labor health

During work performed by healthcare workers, several pressure elements from different sources may impact on keeping optimal conditions for a healthy working environment, and because of the saturation of the sanitary facilities due to the high level of virus infection, the health of these professionals has been obviously affected. We must not forget the efficiency and proper working order of these institutions depend mostly on its professionals' wellbeing, and the conditions in which they are performing their duties are putting at risk the physical and mental health of many of them as they are exposed to different stressors at work. Focusing on aspects related to occupational psychology, we can consider highlighting two groups of main factors which could influence on the possible psychological consequences caused by the pandemic in healthcare workers: lack of resources and heavy workload.

**Lack of resources:** This is a situation that all countries affected by the virus are facing, both material and human resources.

As for lack of material, a high percentage of professionals are getting infected for not having adequate personal protection equipment (PPE) and not using it properly, having to re-use in many occasions equipment which is only recommended for one-time utilization. To get an idea of this, at the beginning of the pandemic in Wuhan, virus transmission reached 29% of healthcare workers in hospitals (3), these high numbers decreased considerably when adequate protection steps were implemented. In Spain, one of the countries most affected by the pandemic, in April, according to Redacción Médica, three out of ten new infected people are healthcare workers (4), this shows the problem's magnitude. Besides the lack of PPEs, we must mention the lack of tests to identify possible cases in hospital professionals, in this way we can isolate tested positive workers to avoid virus propagation. All of this generates fear, uncertainty and insecurity in workers for not knowing if PPEs are protecting adequately and even if they could have the virus and not being aware of it.

Regarding the lack of human resources two elements must be considered. First, medical leaves due to the virus, which is directly related to the lack of equipment mentioned above. Second, the saturation of health institutions. According to data provided by Spain's Ministry of Health at the beginning of April:

In European Union and United Kingdom, in cases confirmed, 30% of persons with COVID-19 required hospitalization and 4% of these were considered critical, defined as requiring mechanical ventilation, or other criteria to be provided with assistance in intensive care unit (ICU) (3).

In this context, it is evident there are not enough human resources to meet current demands, so medical institutions have signed contracts with doctors and nurses being in their last year of residency, as well as with medical graduates without specialization. This last group is formed by professionals who are mostly new in labor environment and might have been overloaded at psychological level because of the situations they have had to face since their little experience which is already a challenge for experienced professionals. The distress suffered in an unknown and new job for them, could generate a negative emotional association to this work environment, becoming an aversive stimulus to which they would not want to be exposed again.

Also, the working hours for many professionals have been modified, having to work more hours than usual, even making double shifts; time needed for resting to guarantee personal wellness and, therefore, a proper job performance. We must not forget fatigue is related to possible accidents, for example handling PPEs that increase the risk of infection. Additionally, the change of shifts could be a problem for family conciliation, increasing even more the pressure they are exposed to.

**High volume workload:** this factor is derived from the first one to a certain extent, but we have decided to take it into account independently as it is something health professionals normally deal with and previous studies have shown it is a factor that affects their health directly, especially in this situation.

Within work overload, there are two different types of overwork: quantitative, which relates to performing excessive tasks during working hours and, in this case, it is related to the saturation of health facilities which have required the reorganization of working days, thus generating physical as well as psychological exhaustion of professionals, as workers not having the opportunity to recover; and the qualitative overwork, defined as to having to cope with excessive demands on their cognitive as well as their emotional skills (5).

Both types of work overload contribute to worker's psychological discomfort but, considering our current situation, the qualitative overload plays a very important part in the consequences which will appear in healthcare workers in the middle term. The situation caused by COVID-19 could generate in workers a feeling of ineffectiveness and helplessness because of this qualitative overwork that they are facing, which in turn contributes to a high emotional load which is already affecting healthcare workers.

As we have said, if the factors described above last in time, they could generate different symptoms at psychological level in a population already predisposed for this type of problems. In fact, it is known that different levels of depression and anxiety are increasing progressively in healthcare workers and are above average of the general population, so it is assumed they could increase for the reasons explained before.

More precisely, one of the consequences caused by these stressors and to which healthcare workers are prone is the Burnout Syndrome (BS), defined as an excessive and prolonged stress whose main components are emotional fatigue causing energy loss, wear out feeling and fatigue; dissociation and, specifically, depersonalization, with regards to an individual's defense upon avoiding those emotions which cause discomfort; and diminish work performance, as work itself loses its previous value (6).

BS is declared by World Health Organization (WHO) as a labor risk affecting person's life quality, compromising individual's mental as well as physical health. Besides, at the organizational level, the worker with BS has not all the capacity to give his patients the healthcare they need, getting the quality of the health services even worse. BS can be identified using the following clinical evidence: social isolation, anxiety, fear, depression, anger, addictions, personality changes, guiltiness and self-immolation, changes in eating habits, substantial gain or weight loss, loss of memory disorganization, problems with concentration and sleep disorders (7).

Due to the effects caused by BS in worker's health at the individual level, as well as the repercussion on health system if it had, as it is expected, a high effect on healthcare workers, the prevention and treatment of BS and its manifestations would be essential for the physical and mental health care in these particular professionals, and the preservation of a high quality health system and attention to patients.

#### Quarantine

In addition to the labor conditions and its consequences quarantine is implemented to stop the expansion of pandemic. Recent history has had situations where quarantine was used as a measure to avoid expansion of contagious disease, such is the case for China and Canada during the outbreak of Severe Acute Respiratory Syndrome (SARS) or in some African countries during Ebola disease. Based on these, we know the psychological consequences caused by this type of isolation.

As stated by Liu, et al. (8) in their study, performed after the SARS pandemic in 2012, for the hospital workers, the post-traumatic stress and depression symptoms associated to the quarantine, can last up to three years after the crisis finalizes. Besides, they add, the healthcare workers placed in quarantine show greater symptoms of post-traumatic stress than the average population. Due to this fact, we consider particularly relevant to focus on this population.

In the systematic review launched by The Lancet (9), other research performed on healthcare workers active during SARS highlights that quarantine can produce a predisposition to suffer post-traumatic stress symptoms (10) or acute stress disorder (11). This disorder, according to ICD-10, is a disorder linked to Post-Traumatic Stress Disorder (PTSD) when an individual suffers in acute and temporary terms -minimum 2 days and maximum four weeks- the symptoms of anxiety as a reaction to an exceptional physical or psychological distress. This experience can be caused by indirect exposition, for witnessing events happening to other individuals, or by being informed about traumatic events that close people have suffered. Consequently, among others, it causes difficulty sleeping, irritation, poor concentration, motion disorders, hyper-surveillance, which could contribute to an increase in burnout.

The individuals with these disorders can show dissociative symptoms caused by the disconnection produced when trying to avoid anxiety by the upcoming event. This means that individuals could feel emotionally senseless or disconnected -as it occurs as consequence of BS-, suffer dissociative amnesia and, in the most severe cases, have the sensation that events are not real.

If not treated in time, the disorder or episode of acute stress could become a chronic PTSD, considered over time, or even a complex PTSD.

PTSD is a disorder, according to ICD-10 (12), characterized by 1) flashbacks or nightmares about the traumatic event which produce terror and strong physiological reactions, 2) avoidance of memories or thoughts related to the event, or to avoid activities, situations or persons related to, and 3) a lasting perception of a current noticeable threat. Due to these symptoms, professionals working in intensive care units (ICU) may not desire to keep working there. If these individuals develop post-traumatic stress, as a self-protection strategy either being aware or unaware, they may not want to return to where it was produced.

Therefore, the psychological consequences derived from the social situation to which the healthcare workers are exposed could not only have implications at individual level, but also increase the burnout already mentioned and may help degrade the health system institution; due to the fact that having professionals with PTSD would decrease human resources either with sick leaves or for not being able to cover certain health services.

## CONCLUSION

Human beings in general have a great resilience capability and adaptability to circumstances. However, as it is known, we require help from other persons to facilitate these processes. It is necessary to consider the skills that healthcare workers require to develop to be able to overcome the circumstances derived from the COVID-19 pandemic, as it has caused a considerable increase in the stress levels they are normally exposed to.

The recommendations published by World Health Organization (13) include one section dedicated exclusively to healthcare workers, where it has suggestions to reduce the impact of the BS consequences described. Besides, another section is specifically focused in one of the consequences of BS: social isolation. The human beings need of counting on others' support, the relevance shown by WHO, added to what has been written in this article, make us consider social isolation for workers of health institutions as a special relevant variable.

As explained, BS has as consequence, generally speaking, social isolation, if we add to this the specific circumstance of quarantine as a measure to stop COVID-19 expansion, and the fear of these workers to infect their loved ones; we face a social isolation higher than expected. Besides, as we have seen, quarantine causes PTSD-especially in this group-, which increases the chances of BS to develop. This scenario could be generating a feedback cycle, developed by social isolation, which requires maximum attention.

Certainly, most of the countries affected by this pandemic, as a result of WHO recommendations, have provided answers to the needs of mental health care. According to these recommendations, psychological assistance to general population becomes highly essential and particularly, to healthcare workers during the pandemic and, at least, up to three years after the event, as highlighted by Liu, et al. (8).

Some experts suggest strengthening the mental health systems and performing stepped care, which means performing, at the beginning, a treatment low in system resources based in stabilization and self-management of patients in need of this attention so as to, when demand decreases, invest more resources and meet the needs of every patient (14).

## ABBREVIATIONS

PTSD: Post Traumatic Stress Disorder
